# Biomarker selection and imaging design in cancer: A link with biochemical pathways for imminent engineering

**DOI:** 10.1016/j.heliyon.2020.e03340

**Published:** 2020-02-03

**Authors:** Joham Surfraz Ali, Noor ul Ain, Sania Naz, Muhammad Zia

**Affiliations:** Department of Biotechnology, Quaid-i-Azam University Islamabad 45320 Pakistan

**Keywords:** MRI, Metabolic pathway, DOPA, PET, SPECT, Cancer, Imaging, Cell differentiation, Metabolomics, Cancer research, Medical imaging, Nuclear medicine, Biochemistry

## Abstract

Malignant cells reprogram metabolic pathways to meet the demands of growth and proliferation. These altered manners of metabolism are now identified as hallmarks of cancer. Studies have revealed tumor cells alter specific pathways such as glycolysis, fatty acid synthesis and amino acid synthesis to support their proliferation. In this review, we provide a theoretical framework to understand metabolic reprogramming and the mechanisms accompanying distorted metabolism to tumor progression. How these alterations will be assisting in cancer diagnostics and advances in standard techniques in marker identification and imagining are also discussed.

## 1 Introduction

Cancer is collectively known as a group of disorders associated with uncontrolled cell division and growth eventually leading to cell invasion and dissemination to the distant sites of the body from their origin. Development of cancer (carcinogenesis), involves various physiological alterations in the cell like dysregulated energy metabolism [1], develops resistance to the cell death, avoiding cell signaling etc. [2]. Unlike normal cells, cancerous cells actively grow even in low serum in masses by evading contact inhibition and adopt round morphology instead of flat and did not establish any connection to the substrate i.e. independent to anchorage. Due to this rapid proliferation, cancerous cells need higher energy source in order to sustain their proliferative potential. Hence, they modify their energy metabolism as compared to normal cells which act as an emerging hallmark of cancer cells i.e. reprogramming of energy metabolism [3]. These modifications from normal cell behavior can be helpful for the prognosis and treatment of these malfunctions.

In this review we emphasize on one of the most important emerging hallmarks of the cancer cells where they exhibit altered metabolism and how these metabolic alterations will be helpful in detection or prognosis of cancerous cells by monitoring their metabolomics and metabolic flux analysis using varying detection techniques opening window for novel therapeutic targets and optimally balanced drug efficacy.

## 2 Cancer; basic alteration of normal metabolism

One of the salient features that differentiates cancer cell from normal is their differential metabolic processes [4]. It is well-documented that altered metabolism is more obvious in cancerous cells where they help cancer cells to endure their sustainability in limited supply of oxygen (hypoxic condition) by facilitating their replication, progression, invasion, and spreading to the distant sites. The demand of oxygen surpasses by oxygen supply in case of cancers and tumors where cells proliferate rapidly. Therefore the gap between cells and blood vessels increases and limits the oxygen supply. Even in the presence of oxygen, tumor cells limit their energy supply mainly to glycolysis, heading to the supposed aerobic glycolysis phenotype. Indeed, the dependence on glycolytic fueling increases the likelihood to hypoxia, a condition that characterizes most tumors. Nevertheless, the level of oxygen required in cancer cells depends on the initial supply of oxygen to the tissue, size and stage of tumor [5,6]. In cancer, increased production of lactic acid was observed due to enhanced glucose import along with higher glucose metabolism and reduction in oxidation of pyruvate. Though glucose catabolism in cancer via aerobic glycolysis has a major contributing factor towards changed metabolism but only this could not be enough to enlighten all necessary alteration in metabolism that sustain the requirement of cell growth [7]. Besides glucose metabolism, increased gluconeogenesis, *denovo* synthesis of fatty acids, glutaminolysis activity, glycerol turnover and pentose phosphate pathway while lower fatty acid oxidation was also important variations in the metabolic processes observed in cancerous cells [1]. We will discuss all of these alterations in cancer metabolism respectively.

### 2.1 Crabtree effect

**Figure 1:**
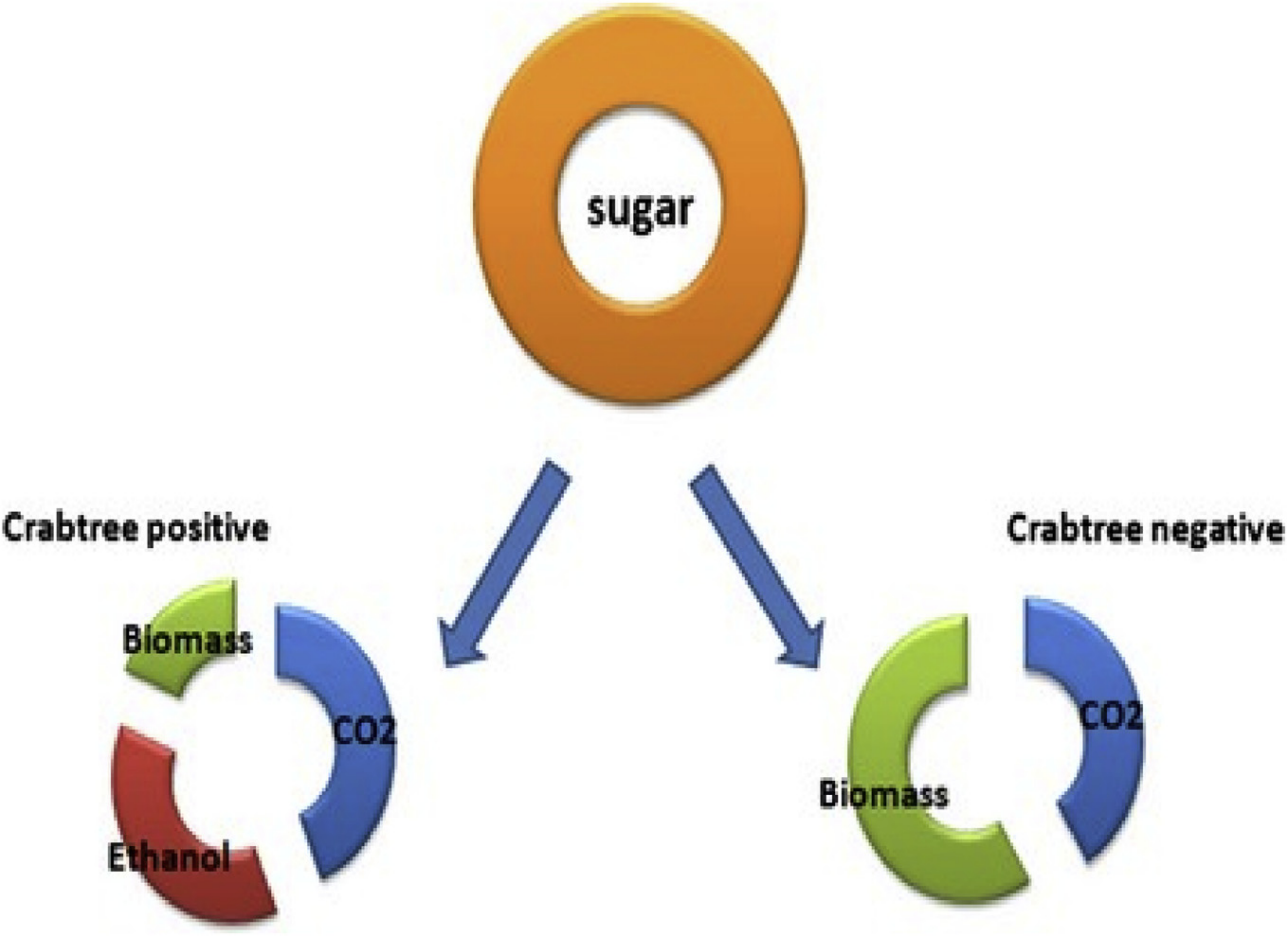
Crabtree positive and negative effect. Crabtree effect demonstrated in yeasts cells with Crabtree-positive and negative cells. It lowers biomass production as a portion of sugar is converted into ethanol. Thus Crabtree-positive yeast cells exhibit increased glucose consumption to attain the same yield of cells in comparison to Crabtree-negative yeast. Though, ethanol acts as a device to reduce and control the proliferation of other competitive microbes.

In 1926, Herbert G. Crabtree proposed that utilization of carbohydrates were done differently in normal and cancer cells [8]. According to his observations, existence of glucose in normal cells results in elevation of respiration without effecting oxygen consumption. While in tumor cells utilization of glucose led to the reduction in oxygen uptake. Respiratory inhibition of cancerous cells by glucose is known as Crabtree effect. This respiratory alteration is vital for rapidly dividing cells like renal cells, embryonic cells etc. [9] And associated with higher glycolytic rate. In addition to this, it is also linked with increase in respiration at first followed by provision of glucose (Figure 1).

### 2.2 Warburg phenomenon

**Figure 2:**
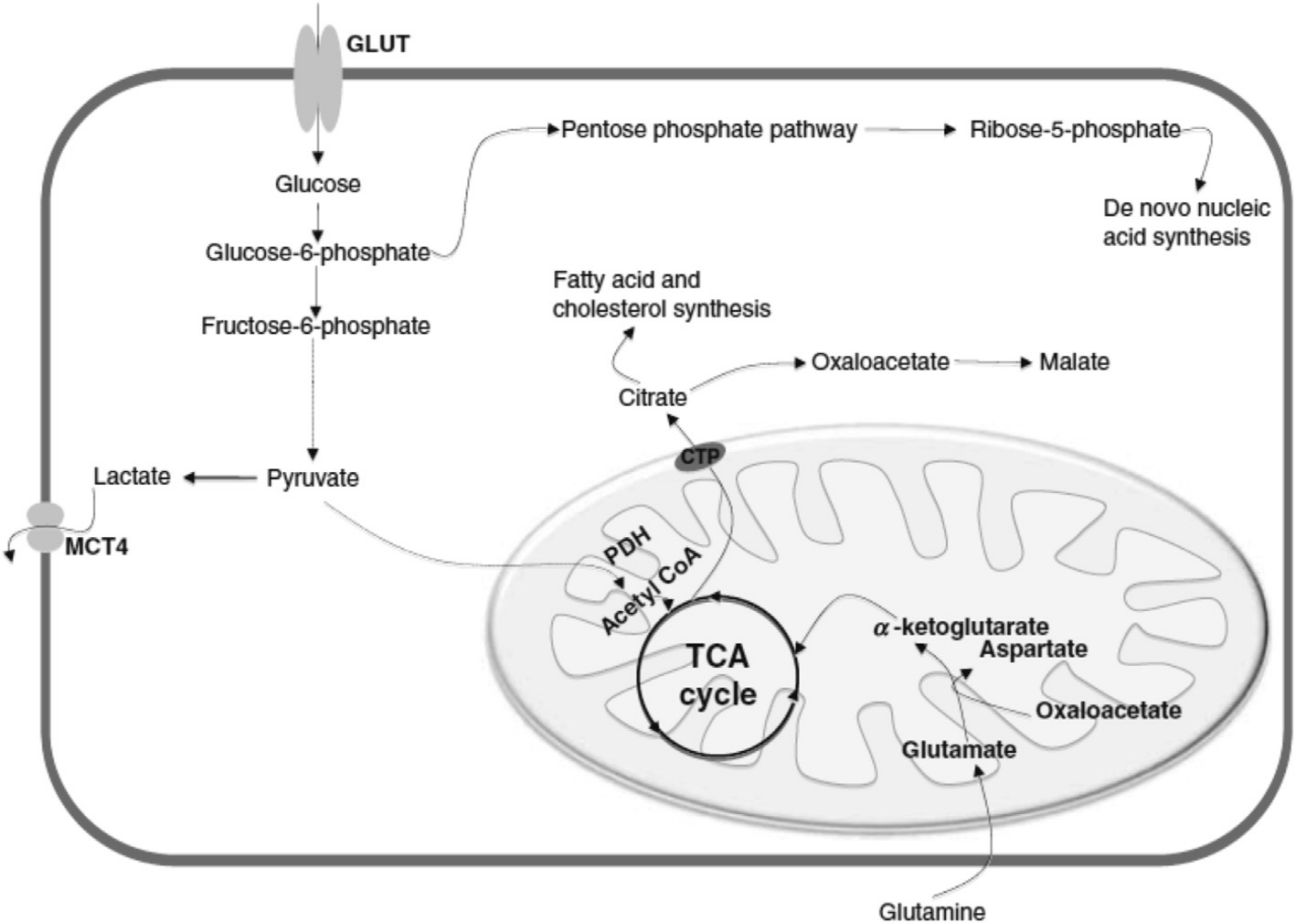
Elevated glycolysis, glutaminolysis, and lipogenesis within cancer cell. Increased glucose uptake powers glycolysis, but because of the inefficient utilization of glycolytic endproducts by the cancer cell, pyruvate is converted to lactate. Cancer cells take in more glutamine that feeds the tricarboxylic acid (TCA) cycle leading to more citrate production. Citrate is transported into the cytosol mediated by citrate transport proteins (CTP). Cytosolic citrate is converted to acetyl CoA that supports lipid and cholesterol biosynthesis.

It is well accepted fact that rapidly multiplying cells demand higher energy in comparison to normal cells. In contrast to this observation, cancer cells attain higher potential for proliferation even extracting energy from a less efficient aerobic glycolytic process known as Warburg phenomenon as explained by Otto Warburg [10–12]. Under constant supply of oxygen, normal cells undergo glycolysis to produce pyruvate and finally oxidize this pyruvate into carbon dioxide via oxidative phosphorylation in mitochondria. In the absence of oxygen, normal cells undergo incomplete oxidation of glucose resulting in production of lactate avoiding mitochondrial respiration [10]. According to Warburg effect, in contrast to normal cells, cancer cells transform glucose into lactate via less efficient aerobic glycolytic process [11]. One of the possible reasons behind this variation is due to the requirement of other metabolic end products which can fasten progression and proliferation of cancer cells during hypoxic conditions and help in avoiding cell death in the presence of cytotoxic molecules [1]. Extensively proliferating cells under hypoxia and activated HIF-1, the electron transport chain is hampered due to absence of oxygen as electron acceptor. The glucose is also redirected from mitochondrial acetyl-CoA-mediated citrate production. An alternative pathway for sustaining citrate synthesis includes reductive carboxylation, considered to depend on a reverse flux of glutamine-derived α-ketogluturate via isocytrate dehydrogenase-2 (IDH2). The reverse flux in mitochondria can be maintained by NADH conversion to NADPH by the mitochondrial transhydrogenase, with the resulting NADPH driving α-ketoglutarate carboxylation. Citrate/isocitrate exported to the cytosol may be metabolized oxidatively by isocytrate dehydrogenase-1 (IDH1), and contributes to the production of cytosolic NADPH [6]. According to Otto Warburg, neoplastic transformation from normal cells initiated due to the irreparable impairment to the mitochondrial respiration. Hence, cancer cells exploit glycolysis to produce 2 ATPs instead of 36 ATPs from utilization of glucose molecules. Alteration of bioenergetics suggested that cancerous cells essentially adopt a mechanism for enhanced import of glucose for regulating their energy demands [12] (Figure 2).

Glycolysis, TCA and electron transport chain all are well coordinated for complete oxidation of glucose molecules. Regulation of the TCA cycle is mainly carried out by the accessibility of the substrate molecules and inhibition was caused by accumulation of products and intermediates produced during TCA cycle. Defects or loss in respiration will eventually result in the accumulation of NADH (nicotinamide adenine dinucleotide), OAA (oxaloacetic acid), succinyl CoA and citrate which are vital regulators of the TCA cycle. Both succinyl CoA and NADH prevent activity of critical enzyme like citrate synthase, isocitrate dehydrogenase, and α-ketoglutarate that are involved in rate limiting of TCA cycle. Furthermore, citrate impedes citrate synthase and NADH hinder functioning of pyruvate dehydrogenase (PDP). Certainly, these alterations in respiration will decrease transformation of pyruvate into acetyl CoA and thuscomprehensive reduction in working of TCA cycle. Therefore, under such situations glycolysis will be more evident type to get energy for cellular processes in cancer cells even in the presence of oxygen [1].

### 2.3 Glutamine metabolism in cancer cells

Glutamine is the utmost copious amino acid present in human serum. Glutamine act as a nutrient and energy source in cancer cells (Figure 2). In proliferating cells, it provides nitrogen, used in the generation of nucleotides, hexosamine and non-essential amino acids [13]. Besides this, actively growing cells exhibit glutamine dependence apart from nitrogen requirement. These cells sustain TCA cycle by resorting intermediate through anaplerosis [14]. The fact that glutamine is a vital carbon source for anaplerosis in majority of proliferating cells via its conversion to glutamate and subsequently into α-ketoglutarate through multiple biochemical reactions collectively known as glutaminolysis [15]. Glutaminolysis involve metabolism of glutamine via a TCA cycle through different mechanism as compared to glucose as it remained unaffected in the presence or absence of oxygen molecule. Entrance of glutamine in TCA cycle is facilitated by two enzymes i.e. glutaminase (GLS) and glutamate dehydrogenase (GDH). Conversion of glutamine into glutamate is carried out by GLS while GDH further transfer this glutamate into α-ketoglutarate. Higher activity level of basal GLS was observed in breast cancer cells and fibroblast. In addition, this enzyme was frequently upregulated in MYC transformed cells [7]. No requirement of exogenous glutamine has been detected in cancer cells due to enhanced activity of glutamine synthetase. Yet, some other amino acids are also used up by the cancerous cells and disparity in expression of certain enzymes and amino acid transporters (AATs) involved in catabolism of the amino acids in many cancers [16]. In majority of cases, glutamine is utilized by cancerous cells to produce lactate and alanine by the involvement of malic enzymes and led to the generation of NADH and oxaloacetate (OAA) in an anaplerotic reaction [17]. The oxaloacetate (OAA) enters the TCA cycle and consumed in the generation of citrate. Export of citrate into the cytosol is carried out by special transport proteins known as citrate transport proteins (CTPs) due to the truncation of citrate cycle in cancerous cells. Higher activity of CTPs has been observed in cancer cells to facilitate transportation of citrate. This cytosolic citrate is then used for the synthesis of lipids in lipogenesis process. Consequently, metabolism of glutamine acts as a source of both NADH and citrate for enhanced rate of lipogenesis in cancerous cells. In addition, carbon derived from glutamine in TCA cycle being part of aspartate which in turn used in the formation of nucleotides and amino acids like aspartate and arginine [17]. Glucose and glutamine are the most abundant nutrients for producing energy and building blocks in normal and tumor cells. Increased glycolysis in tumors, the Warburg Effect, is the basis for ^18^F-FDG PET imaging. Cancer cells can also be genetically reprogrammed to use glutamine [18]. 5-^11^C-(2*S*)-glutamine and ^18^F-(2*S*,4*R*)4-fluoroglutamine may be useful complementary tools to measure changes in tumor metabolism.

### 2.4 Lipid metabolism pathways

Cancer cells also exhibit altered lipid metabolism and higher biosynthesis of *de novo* fatty acids in addition to aerobic glycolysis and increased glutaminolysis (Figure 2). Literature review showed *de novo* synthesis of fatty acids in cancerous cells regardless of the extracellular lipids concentration [1]. This might be due to the requirement of fatty acids for membrane synthesis and production of signaling molecules to activate cell proliferation resulting in cancer. Numerous metabolic products produced by metabolism of lipids are elevated in cancerous cells [19]. Synthesis of fatty acid needs acetyl-CoA and NADPH and integration of carbon and redox metabolic pathways. Generally for fatty acid synthesis glucose act as an important source of acetyl-CoA in majority of cultured cells [17,20]. Glutamine and acetate offers an alternative carbon sources for acetyl-CoA when dysregulation in mitochondrial functions and lower concentration of oxygen results in lessened supply of acetyl-CoA from glucose [21, 22, 23, 24]. In some cell lines leucine act as a source of acetyl-CoA through it metabolism [25]. Reduction of dihydroxyacetone phosphate to glycerol-3-phosphate efficiently supply molecules that are vital for the synthesis of both phospholipids and triacylglycerols while 3PG-derived serine help in the synthesis of phospholipid [26]. Moreover, citric acid synthesized from TCA cycle also assists as a precursor molecule for fatty acid synthesis endogenously. Citric acid is transported to the mitochondria and transformed into malonyl-CoA via ATP citrate lyase (ACYL) and acetyl-CoA-carboxylase (ACC). Fatty acid synthase (FASN) then convert this malonyl-CoA into palmitate [27]. FASN is a multifunctional enzyme and acts as an important enzyme involved in the synthesis of fatty acids from carbohydrates in cancerous cells. Normal cells do not express FASN as they depend on circulating lipids while constitutively overexpressed in majority of cancers especially in excessively proliferating tumors. Concurrently, its expression is a primary incident towards cancer development and its level of expression is linked with cancer progression from early to late stage and helps in prognosis and diagnosis of cancer [1].

### 2.5 Citrate metabolism by prostate glandular epithelial cells

It is well recognized that significantly decreased level of zinc and citrate is a typical feature of the cancer as compared to normal prostate cells. Citrate itself is a metabolic marker in different cancers such as prostate. These biochemical alterations arise in early cancer's progression. The transition in metabolism of the prostate gland is distinct from other body cells [28]. Progression of prostate cancer is linked with an early variation in metabolism from normal citrate secreting epithelial cells to citrate oxidizing malignant cells as well as zinc accumulating prostrate epithelial cells to zinc deficient cells [29,30]. This metabolic transformation is due to the reduced expression of zinc transporters which consequently results in reduced zinc level [31,32]. Additionally, malignant prostate tissue essentially never attain the elevated levels of citrate and zinc present in normal peripheral zone. Reduction or deficiency of zinc consequently responsible for two significant effects that are metabolic effect and other is growth effect. In metabolic effect; inhibition of m-aconitase activity by zinc does not take place under reduced zinc level and oxidation of citrate occur through Kreb's cycle which greatly effect bioenergetics of the cell. This leads to higher energy production i.e. 36 ATPs/glucose molecule which is quite higher than 14 ATPs/glucose molecule through aerobic oxidation of glucose [1]. By this revenue of energy generation cancer cells are bioenergetically more efficient than the specialized epithelia of the normal prostate cells. This mode of energy production delivers the extra energy to the malignant cells to achieve carcinogenesis required to attain malignancy. This phenomena of citrate oxidation was distinct from the general metabolism exhibited by tumor cells in which they extract energy by enhanced aerobic glycolysis [33, 34, 35]. While growth effect due to reduced zinc is from the exclusion of the apoptogenic influence of zinc which in combination with the metabolic effect allows the proliferation and growth of the cancer cells. These adaptions never proceeds in the presence of higher accumulation of zinc that's why zinc accumulating, citrate producing cells are absent in prostate cancer [36].

## 3 An ideal biomarker

**Figure 3:**
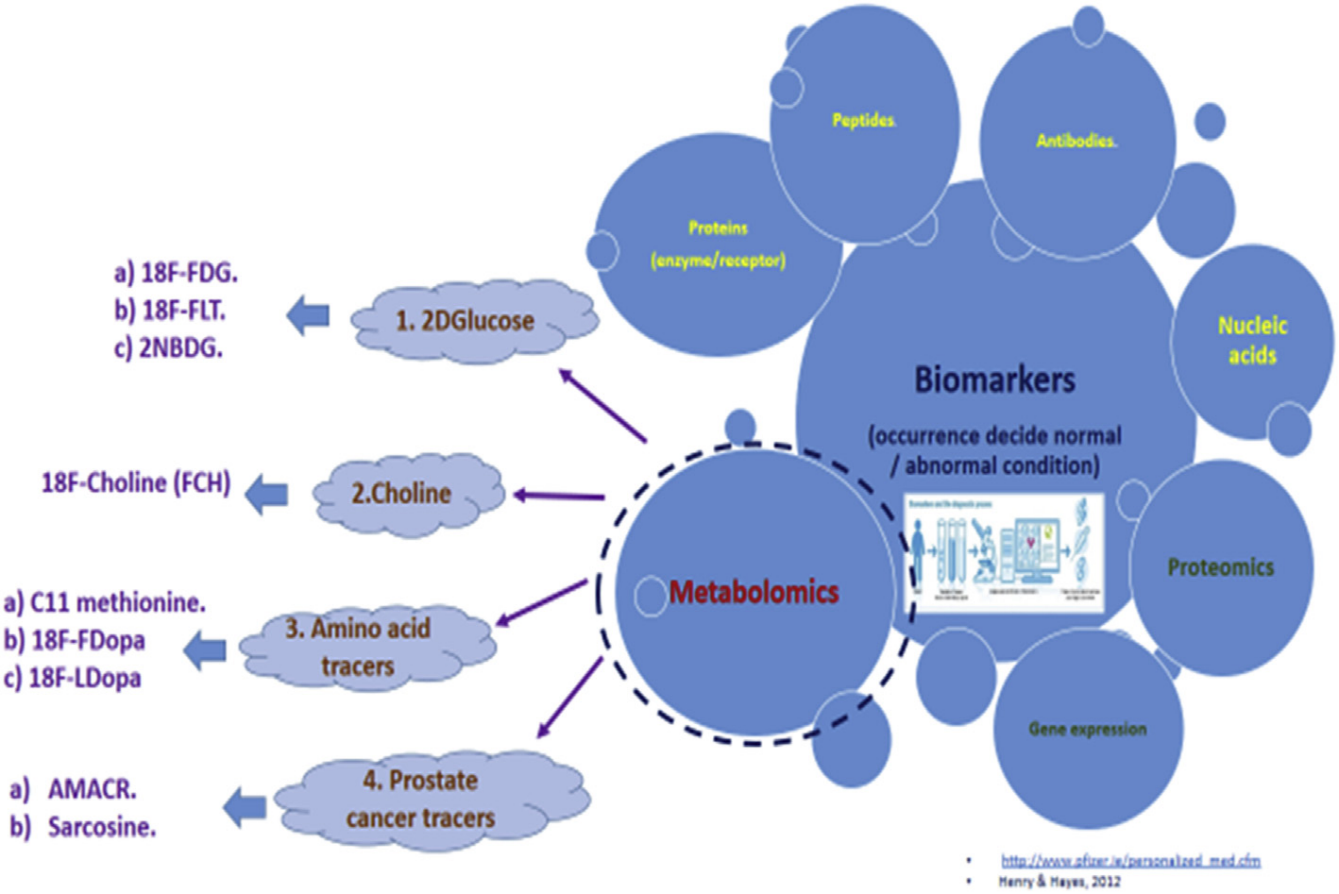
Biomarkers associated with metabolic pathways including glucose, lipids and amino acids.

National Cancer Institute (NCI) defined biomarker as “a biological molecule found in blood, other body fluids, or tissues that is a sign of a normal or abnormal process, or of a condition or disease,” such as cancer. A biomarker normally distinguishes a patient affected with disease from a person with no disease. The variations can be due to numerous causes and factors, involving mutations at germline or somatic level, transcriptional changes, and posttranslational alterations. There is great variety of biomarkers, including proteins (an enzyme or receptor), nucleic acids (microRNA or other non-coding RNA), antibodies, and peptides, among other types (Figure 3). It can also be a group of modifications, such as gene expression, proteomic, and metabolomics signatures [37].

A biomarker must be indicated to associate with a concerned result, like progression of disease, relapse, or persistence, thus beneficial for diagnosing and scrutinizing a disease. Numerous statistical evaluations, with various variables should validate that the biomarker is able to predict the appropriate stage or grade regardless of the features commonly available [38].

An ideal biomarker should be rapid, reliable, cost-effective, and measurable in an available biological fluid or clinical sample when successfully modulated by combining effective imaging technique and measuring possible risk assessment of a particular disease which is readily interpretable by a clinician. Its expression should be significantly increased (or decreased) in the related disease condition, and no overlap should exist in the levels of outcomes between healthy control subjects and untreated patients [39].

Early diagnosis and correct staging are the fundamentals of a successful cancer therapy. By 2030 deaths due to cancer is expected to rise from 7.4 million in 2004 to 23.4 million [40]. Physicians typically depend on variation in size and location while detecting and examining malignancies, measures which are belated signs of ailment. Although biopsies offer the maximum information about a cancer, but this procedure is invasive for examination. Furthermore, specimens of biopsy got issues such as sampling error hence the complete tumor load cannot be assessed. Frequently, a solid tumor big enough to visualize and biopsy is very varied, making sampling with the needle even more difficult. Therefore, non-invasive molecular imaging of a cancer before, during, and after therapy possibly augment our perception and knowledge of cancer biology. Moreover, contribute towards realization of personalized therapy through early diagnosis and monitoring [41]. Few such affective agents are discussed below (Table 1).

**Table 1:** Metabolic biomarkers and their significance.

Biomarker	Analogue	Targeted Metabolism	Cancer	Significance	Limitations
Glucose	2DG	Aerobic glycolysis	Almost all cancers	• Oxidative stress, • Glycosylation inhibition, • Facilitates apoptosis,	• False positive results due to frequent uptake by normal and inflamed cells.
	18F-FDG	Glycolysis	• Colorectal, Lung, Head, • Neck, Breast, Gall bladder, Lymphoma,	• Detecting malignant lesions, • FDA approved, • Efficient early diagnosis,	• Difficulty in detection of inflammation lesions,
	18F-FLT	Thymidine kinase (DNA synthesis, cell cycle/nucleotide synthesis)	• Extra cranial tumors • Lung, Melanoma, Laryngeal, Soft tissue,	• Better imaging contrast than FDG, • Promising tracer for gliomas,	• Poor spatial resolution
	2-NBDG	Glucose uptake	• Oral neoplasia, Malignant cells,	• Submicron resolution image, • Significant substitute of radiopharmaceuticals, • Easy production, • Easy local topical delivery,	• Uptake by benign lesions and penetration depth
Choline	^11^C-choline	Lipid synthesis	• Brain, Lungs, Colon, • Esophageal, Bladder, • Prostate,	• Used to image breast, head and hepatocellular carcinoma,	• Insufficient diagnostic accuracy in staging the disease, Allergic reaction, • Radiation risk
	^18^F-choline (FCH)	Membrane lipid and protein	• Brain, Prostate,	• Longer half life	• Less specific and sensitive than MRI
Androgen receptor	^18^F-FDHT	Protein synthesis, (antigen)	• Prostate, • Breast,	• Noninvasive approach for tumor study,	• reduced affectivity when administered along testosterone
Amino acid	C11-methionine	Amino acid transport, Polyamine synthesis, Protein synthesis,	• Brain, Melanoma, Endometrial, Lymphoma,	• Effective Monitoring tool for therapeutic effect, • Identifies tumor recurrence, • Differentiate b/w neoplastic and non-neoplastic origin of hemorrhage,	• Binding to protein, • Accumulation in pelvic bone, esophagus, pancreas, endometrium etc,
	18F-F-DOPA	Ambiguous mechanism but associated to Neutral amino acid transporter	• Brain tumor	• Monitor striatal dopamine pathway, Identify movement disorders, Longer half-life,	• Confusion in its accumulation either due to dopaminergic reaction or due to tumorigenicity
	18F-OMFD	Neutral amino acid transporter	• Brain lesions	• Similar kinetic profile as 18F-FDOPA,	• Expensive due to complicated labeling procedures.
	18F-L-DOPA	Catechol amines biosynthesis	• Neuroendocrine, Laryngeal, Melanoma,	• Effective for solid neoplasms	• Expensive due to complicated labeling procedures.
Glycoprotein	PAP PSA	Basal cell layer disruption causing PSA leakage,	• Prostate,	• Used in prostrate cancer prognosis. • Used as staining agent to determine the malignant cells being metastasized	• Less sensitivity, • Detection of proper stage issues, • False positive results
AMACR		Fat metabolism	• Prostate,	• Growth promoter, • High sensitivity and specificity,	• Feasibility issues, • Humoral response,
Amino acid (glycine derivativ)	Sarcosine	Amino acid	• Prostate,	• Malignancy of prostate cancer,	• Still under clinical trials,

### 3.1 2-Deoxy-D-Glucose

Cancer cells are distinguished from normal cells via altered metabolism of glucose i.e. increase in aerobic glycolysis and it is known as Warburg effect as described above (Figure 2). Although, there is adequate amount of oxygen, yet glucose is converted to lactate. Intervening with this activity may be an effective approach to initiate death of cancer cells since these cells are heavily dependent on glucose metabolism for survival and propagation. An analog of glucose known as 2-Deoxy-D-glucose (2DG), aims at glucose metabolism to deprive cancer cells of energy. Furthermore, 2DG upsurges oxidative stress, N-linked glycosylation inhibition, and induction of autophagy. Cell growth is effectively slowed down by 2DG and potently facilitate apoptosis in specific cancer cells. Even though 2DG has limited therapeutic effect in numerous types of cancers, it may be combined with other therapeutic agents or radiotherapy to exhibit a synergistic anticancer effect [42]. But in this review, we will be mainly focusing on the detection of cancer via 2DG and its analogs. Since it is known fact that cancer cells utilize glucose and convert it into ATP, hence it has become the base of tumor imaging with fluoro-deoxy-glucose (FDG) positron emission tomography (PET) exhibiting increased uptake of glucose in tissues with tumor as compared to normal [43]. To image glucose uptake activity down to single-cell resolution, glucose analogues that are labeled with fluorescent dyes have thus been developed (for use in fluorescence microscopy, with optical resolution).

#### 3.1.1 Fluorine-18-fluorodeoxyglucose (18F-FDG)

18F-FDG is an analogue of glucose, is processed and metabolized just like glucose. Glucose transporters present on the cell membrane transport FDG across cell membranes like a glucose molecule and is phosphorylated to FDG-6- phosphate via enzyme action but it cannot go under further glycolysis in comparison to glucose-6-phosphate and hence stuck and fixed inside the cell [44]. PET has been suggested as non-invasive imaging technique to evaluate the magnitude of disease in cancer patients. As 18F-FDG is a glucose analogue, thus this radioactive molecule can be very beneficial in detecting and identifying malignant lesions exhibiting high glucose metabolism [45,46].

FDG is the only radioactive tracer that has been approved by FDA and is used in almost all cancer types, diagnosis, staging, restaging, and in observing response to cancer treatment. The stage at diagnosis is the most powerful predictor of survival, and treatments are often changed based on the tumor stage; therefore, accurate staging is critical. Restaging is performed after the treatment to detect residual tumor or suspected recurrence, and to determine the extent of a known recurrence or distant metastasis. The power of FDG-PET is fully demonstrated by detecting metastasis through a single whole-body PET scan. The purpose of using PET for disease monitoring is to provide an early assessment of therapeutic response or treatment refractory disease [47,48].

##### 3.1.1.1 FDG and cancer imaging

Numerous studies have assessed the diagnostic accurateness of 18F-FDG-PET in various cancers including colorectal [49], esophagus [50], gall bladder [46], head and neck [51], seminoma [45], lymphoma [52], non-small cell lung and breast cancer [53]. In all these cancers, FDG imaging has been successfully used for the early assessment of the cancer and furthermore treatment response.

Although, tumor imaging via FDG-PET is a promising technique but images are necessarily to be reviewed carefully to circumvent false-positive elucidations. Moreover, tumor cells and normal cells like brain and heart cells utilize huge quantities of glucose due to high metabolic need. Also, body uses for curative and detoxification, hence kidneys and bladder both comprise a byproduct of this method and imaged incidentally. Thus, for early detection of persistent or recurrent disease, a PET tracer other than FDG, with no increased uptake in inflammatory lesions, would be desirable, such as 18F-fluorothymidine (18F-FLT) which reflects cellular proliferation rather than less specific increased glucose metabolism [54].

#### 3.1.2 3-Deoxy-3-18F-fluorothymidine (18F-FLT)

18F-FLT is the thymidine analog, developed as a PET tracer for *in vivo* imaging of cancer lesions. This tracer is maintained in tissues that are proliferating via thymidine kinase activity [55]. Thymidine kinase-1 (TK1) is an enzyme which is expressed in DNA synthesis phase of the cell cycle. Uptake of 18F-FLT has been shown to exhibit the activity of TK1 [56]. In proliferating cells, TK1 is highly active as compared to non-dividing cells in which activity of TK1 is low. TK1 phosphorylates FLT forming a molecule of FLT monophosphate with a negative charge, results in trapping and accumulation of radioactive tracer inside the cell. It has been examined in several extracranial tumors, including human lung cancer, colorectal cancer, melanoma, lymphoma, breast cancer, laryngeal cancer, and soft-tissue tumors. A comparative study was done between FDG and FLT in brain tumor and it has shown that image contrast of FLT was better than FDG and inferred FLT as a promising tracer in gliomas [57].

#### 3.1.3 2-(N-(7-nitrobenz-2-oxa-1,3- diazol-4-yl) amino)-2-deoxyglucose—2-NBDG

It is another most commonly used fluorescent probe of 18FDG for imaging purpose. Although, tagging glucose molecule with luminous dyes is a feasible technique for tumor imaging and detection but it modifies both physical and chemical characteristics of the molecule. Additionally, the labelled fluorophores are normally bigger than the glucose itself. Hence, mostly fluorescent glucose analogs show troubled performances and interactions which are not desirable in cells and tissues, i.e., interrupting the correct glucose uptake activity and delivery [58].

2-NBDG is a fluorescent glucose analog that undertakes a parallel pathway of uptake and metabolism to 18FDG and accumulates specifically in malignant cells. 2-NBDG accumulation was shown to be higher in cancerous cells than in normal cells, in preclinical imaging studies [53]. 2-NBDG has advantages over 18FDG including; 1) Widefield images as well as submicron resolution images can be obtained, 2) combining widefield imaging and targeted high-resolution imaging is perfect for not only detection but also for real-time mapping of tumors, 3) non-radioactive analogs are striking substitute to radiopharmaceuticals, for topical applications 4) easily produced via commercially available reagents, decreasing the limits related FDG agents and 5) molecular weight of 2-NBDG is just 330 Da, it is suitable for local topical delivery with no need for IV injection [59]. Due to potent exposure to radiations is a major limitation in using radioactively labeled 2-DG. However, 2-NBDG resolves this concern *via* optical contrast with minimal toxicity and rapid clearance to urine [60]. Numerous reports have shown that 2-NBDG is transported to tumor cell lines through the glucose transporters. A study has been conducted for imaging of oral neoplasia and it has shown 2-NBDG provides image contrast in both widefield and high-resolution fluorescence imaging modalities, highlighting its potential in early detection of oral neoplasia [61].

### 3.2 Choline

There tend to be three major purposes associated with Choline and its metabolites i.e. acetyl choline synthesis, structural integrity and signaling within cell membranes. It is mainly linked to lipid metabolism and is also a source of methyl that mainly participates in the biosynthesis of S-adenosylmethionine biosynthesis of S-adenosylmethionine (SAM). (1,2). Choline is either up taken using dietary supplements or synthesized by methylation of phosphatidylethanolamine using N-methyltranferase to produce phosphatidylcholine within liver (3) or sometimes gain its methyl groups from betaine (4). It is an interconnected pathway where generally methionine is either formed from methyl groups from folate or betaine that usually gets it from choline. Alterations in any one pathway gets compensated by another one and even if none works and insufficiency of methylation procedure occurs Homocysteine acts as a precursor (4).

A precursor of phosphatidylcholine and a key component of membrane lipid, choline is highly activated during proliferation of cells and active in synthesis of membrane lipids and proteins. Consequently, tumor cells utilize choline in great quantities by tumor cells. Thus, carbon-11 choline (^11^C-choline), an oncological PET tracer was developed for assessment of a diverse range of malignant tumors involving brain tumors, lung cancer, esophageal cancer, colon cancer, bladder cancer, prostate cancer, and many other cancers. Uptake of ^11^C-choline is considerably higher in malignant tumors as compared to benign, better associated with the level of FDG deposition in the lesion. Also, it has no background activity in comparison to FDG, due to excretion through urinary tract hindering FDG-PET examination. Mostly studies have been done on ^11^C-choline imaging include prostate cancer and brain tumor [62]. But it has been reported in imaging of breast cancer, head and neck cancer and hepatocellular carcinoma. Another isotope of choline, ^18^F-choline (FCH) has much longer half-life in contrast to that of C-11 and shown to be advantageous [44].

### 3.3 Amino acid based biomarkers

Formerly 18F-FDG was used for imaging brain tumors using PET [63] but the diagnostic limitations i.e. less specificity of 18F-FDG PET and high physiologic metabolic rate of glucose by normal brain cells, the detectability of low grade tumors and recurrent tumors got difficult [64]. In addition to all these limitations, there is also a possibility of insolence of the tracer by benign lesions like inflammation [65] and extensive distribution of it in the brain [66]which appealed for the use of proxy PET tracers for tumor imaging and detection. Amino acid based PET tracers and their analogs enact another class of attractive tumor imaging agents because of their higher uptake in tumor while lower in normal brain tissues [67]. Among which the best studied are 11C-methionine (MET; 11) and 18F labeled aromatic amino acid analogs L-Dopa, F-Dopa, O-2-18F-fluoroethyl-L-tyrosine and many more like these similar in affectivity to that of MET [68].

Majority of brain tumors show an elevated uptake of amino acids in comparison to normal brain [66] mainly including naturally occurring L-[11C] methionine (MET), L-[11C] leucine, and L-[11C] tyrosine and synthetic amino acids such as [11C]1-aminocyclobutane-1-carboxylic acid, [11C] amino isobutyric acid and [11C]1-aminocyclopentane-1-carboxylic acid. Radioisotope labelled (123I-labeled) amino acids are also used extensively for oncology [67,69]. Amino acids are mainly taken up through energy independent L-type amino acid transporter system and sodium dependent transporter system [69]. They are detained within tumor cells due to their incorporation into proteins and higher metabolic activities than normal cells [70]. Along with enhanced energy production. Cell division and protein synthesis over expressed transporter system is also a characteristic of malignant transmogrification [71]. Despite of their use in detection of tumors L-[11C] leucine, [18F] fluoro-α-methyl tyrosine L-[11C] MET and [18F] fluoro-tyrosine [65,69] but still they haven't got FDA approval. Their detection and imaging is mainly associated to the amino acid transporters and their incorporation into proteins usually which is a small fraction. [11C]MET has expanded use in detection of lung, breast, neck, brain and head cancer along with lymphomas [PubMed]. Due to its tendency to cross blood-brain barrier and incorporation into RNA, DNA and lipids and its sensitivity to radiotherapy in comparison to FDG strengthens its usage as a monitoring treatment of cancer.

#### 3.3.1 C11 methionine (11C MET)

The occurrence of methionine correlates to the growth rate of cell facing abruption, proliferative activity of tumor or on the histological grade of cancer (brain gliomas, breast and lung cancer). Apart from cancer cells its high accumulation can also be observed in normal pancreas, bone marrow, salivary glands, pelvic bones, endometrium, bowel and liver. It has high tendency to bind to protein (90%) [71]. It's an effective amino acid based biomarker used as diagnostic aid and radioactive (neoplastic disease). In majority of brain tumors its used as diagnostic and detection tool using positron emission tomography. Positron emission tomography (PET) using methionine C 11 (MET-PET) as an agent to locate and differentiate primary brain tumors and to monitor tumor irradiation therapeutic effect [72].

Patients with tumor surgical excision in whom neurological symptoms restore, MET-PET provide early detection especially in case of tumor recurrence. Radiotherapy treated ones with cerebral tumors MET-PET in amalgamation with PET using flurodeoxyglucose F 18 can convalesce the accuracy of differentiation of recurrent brain tumor from radiation necrosis. MET-PET is also useful in extricating between neoplastic and non-neoplastic origins of intra cerebral hemorrhage [65]. Neoplastic hematomas experimental cases represented enhanced methionine C 11 uptake that comply with magnetic resonance (MR) images and contrast-enhanced images [65]. For the detection and staging of several carcinomas like lung, endometrial, lymphoma, breast, head, lymphoma and neck tumors MET-PET can be used. Amino acid transport, transmethylation, protein synthesis, trans-sulfuration, as a precursor of S-adenosyl methionine (biologic methyl group donor) and polyamine synthesis pathway all procedures contribute in amino acid accumulation of methionine acting as prime principle of its usage as a diagnostic marker [73] Among all these amino acid active transport is the most prevalent as these are accentuated in malignant tissues. C11 is used as a tracer to measure the excursion of methionine using PET [71]. The occurrence of methionine is correlated to the growth rate of cell facing abruption, proliferative activity of tumor or on the histological grade of cancer (brain gliomas, breast and lung cancer). Apart from cancer cells its high accumulation can also be observed in normal pancreas, bone marrow, salivary glands, pelvic bones, endometrium, bowel and liver. It has high tendency to bind to protein (90%) [71].

#### 3.3.2 F-Dopa

The 18F-FDOPA metabolite 3-O-methyl-6-18F-fluoro-L-DOPA (18F-OMFD) latterly proved to be useful for brain tumor imaging by PET [74] used for evaluating movement disorders (Parkinson's disease) in assorted patients by monitoring the intrinsic striatal dopamine pathway [66,75]. It is also an amino acid analog and is mainly taken up at blood-brain barrier in normal brain tissues by neutral amino acid transporter pathway while for cancer/tumor specific mechanism of 18F-FDOPA uptake is still uncertain while its intimately akin amino tracers i.e. 18F-OMFD, O-2-18F-fluoroethyl-L-tyrosine and 11C-methionine follow the neutral amino acid transporter pathway [76,77]. A similar kinetic profile for 18 F-FDOPA uptake is mainly observed for normal and tumor cells but there occurs a significant contrast enhancing difference between tumors and radiation necrosis on 18F-FDOPA PET scans [74]. It has diversified clinical application due to its longer half-life [78,79]. It's mainly detected in the substantianigra, caudatum and putamen nuclei of brain, complementary in structures and have a trivial embryologic origin [80].18F-DOPA is a prevalent biomarker proposed for the diagnosis of primary brain lesions, particularly of its high uptake in tumors in contrast to its minute uptake in normal brain tissues, other than basal ganglia [81]. Experimentally its proved that 18 F-FDOPA is superior to 18F-FDG in case of recurrent low grade gliomas even using MRI [82]. Encircling its extensive use after surgery and radiotherapy [83]. The usefulness of 18F-DOPA is momentous in the management of currently diagnosed and recurrent brain tumors [82,84,85]. 11C-MET and 18F-FDOPA depicts matching image patterns regarding size shape and image assessment of tumor.

Various publications have demonstrated that glioma cells are commonly found beyond the area of contrast enhancement in MRI, especially in tumors with high grade [86,87]. The enhancement of contrast media in MRI depends on blood-brain barrier permeability that can be affected during necrosis induced by chemotherapy or radiotherapy. Moreover, necrosis by itself can show meaningful contrast enhancement. The relatively long half-life (110 min) of ^18^F-DOPA makes it suitable for transportation to centers with no cyclotron facility on site, allowing for more ‘flexible’ imaging timings, and offers the possibility of late images, which are very important in some cases. The relatively long half-life of the tracer also allows for late specific spot images that can be very useful in case of interpretative doubts (e.g. activity in the urinary system or in the bowel).

#### 3.3.3 L-Dopa

A pyridoxal-5′- phosphate–dependent enzyme, L-DOPA decarboxylase (DDC EC 4.1.1.28) concur a key role in catecholamines biosynthesis that alters the gene expression and synthesize dopamine (significant neurotransmitters in CNS) that by decarboxylation of L-3,4-dihydroxyphenylalanine (L-DOPA) convert it to dopamine. L-Dopa consist of 2 homologous subunits with a molecular weight of 50 kDa approximately purified and characterized from human kidney [88] and pheochromocytoma [89]. In normal individuals its neurotransmitter biosynthetic function is notable in a course of peripheral organs like lungs, gastrointestinal tract, pancreas, liver, kidney and adrenals while the sole purpose still needs to be determined. The tendency of DDC (dopamine decarboxylase) to enzymatically convert aromatic amino acids in extraneuronal tissues as well [90] that testimonies its involvement in cell proliferation and survival [91]. There occurs 15 exons in DDC gene integumenting 85 kb genomic DNA [92]. The diagnosis and prognosis of disparate human revulsion is linked to alterations in messenger RNA expression patterns of DDC that make it an effective neuroendocrine tumor marker [93]its dispassionate efficiency has been latterly verified in other solid neoplasms as well as laryngeal cancer [94]. Even the malignant melanoma also possess a special biochemical pathway for the conversion of L-Dopa to melanin mainly catalyzed by tyrosinase that is restrained in normal and malignant melanocytes paving way for design of special diagnostic agents incorporating melanin precursors [95].

Several other radiopharmaceuticals recently engaged or under consideration in molecular imaging with PET/CT are 18F-fluorothymidine (18F-FLT) [69], 18F-fluoroethyltyrosine (18F-FET) [67], (18F or 11C) choline [77], and18F-fluoromisonidazole (18F-MISO) [67]. Even if typified by varying metabolic pathways, all these tracers have a significant low rate of dissemination in brain structures that avows the identification of functional disorders of brain tumors and metastasis.

### 3.4 Biomarkers for prostate cancer

The second prime cause of male cancer related deaths is Prostate cancer (PCa) in the western world [96]. Prostate Cancer Foundation of Australia statistical analysis manifest the equal ratio of deaths of women from breast cancer to that of men from PCa [97]. Diagnostics associated to PCa has perilously increased in the past decade to the establishment of PSA (prostate specific antigen) test and ageing population [98]. Several treatments formerly used for prostate cancer include digital rectal examination (DRE), biopsies, PSA and histopathological staging [99]. Each having its own pros and cons. Risks associated to conventional methodologies paved way for use of emerging ideal biomarkers (genetic, metabolic) for diagnostics, prognosis and treatment to efficiently extricate between healthy patients, castration resistant prostate cancer (CRPC), re- occurring metastatic prostate cancer, benign prostatic hyperplasia (BPH) and clinically insignificant cancer (indolent PCa).

#### 3.4.1 Prostatic acid phosphatase and prostate specific antigen tests (PAP and PSA)

A glycoprotein dimer, prostatic acid phosphatase (PAP) is a glycoprotein dimer formed principally by the prostate and originally used as a serum biomarker for the detection of metastatic PCa [100] Low sensitivity for localized disease detection [101]caused its replacement from prostate specific antigen (PSA) test. PSA is secreted by epithelial cells of prostate and is a 33 kDa serine protease (kallikrein-3). Normal prostate observe PSA secretion from prostatic epithelium into secretory ducts subsidizing into seminal fluid while in case of PCa, basal cell layer disruption cause PSA “leakage” into circulation causing elevated PSA levels within serum [102]. Though the elevated PSA levels may be due to non-cancer related BPH, diet diversification, environment, prostatitis and medications [103] along with that stage identification of PCa, while metastatic PCa accurate therapeutic identification and decision gets difficult [104]. Despite the success stories associated with decrease in PCa it's uncertain that whether it's due to PSA screening tool or current PCa treatments [105]. The unnecessary biopsy investigations is mainly due to high false positive rates of PSA test and cause macabre side effects like urinary incontinence, erectile dysfunction and serious infections [106]. All these issues inquire a need for a novel and effective diagnostic and prognostic marker for PCa. Slow growing nature of symptomless PCa binders its aboriginal detection [107]. The low impact detection of these tests caused a focus on new diagnostic test capable of former detection of advanced disease, prediction of metastatic disease, distinguishing organ-confined versus extra-capsular invasion and recruitment to active surveillance and also more accurately inform clinical decision-making to avoid unnecessary treatments. Initially various developments have polished the PSA test to escalate the diagnostic accuracy of different forms of PSA [108].

Several other biomarkers are also contributing in their diagnostic efficiency against prostate cancer.

#### 3.4.2 α-Methylacyl coenzyme A racemase (AMACR)

An enzyme (AMACR) confined to peroxisomes, involved in fat metabolism and also function as a growth promoter but does not depend on androgens in case of prostate cancer [109,110] experimentally also observed to depict overexpressed production in prostate cancer assisting diagnosis [111]. In normal cells its considerable function is β−oxidation of branched chain fatty acid molecules within peroxisomes [112]. While in prostate cancer their production escalates to 9 folds approximately [113] that suggest itsuseas detection marker [113]. It also facilitates analysis and interpretation of prostate needle biopsy samples, usually confronting [114] and helpful in detecting serum autoantibodies released in response to AMARC tumor associated antigen especially when mingled with PSA screening. It possesses high sensitivity and specificity as compared to PAP and PSA. Certain limitations associated with it; the feasibility of endogenous AMACR antibody and humoral response as a result of other cancers in autoimmune disease suffering patients [38]and patients suffering from urological disorder [115].

#### 3.4.3 Sarcosine and androgen receptor (AR)

A natural amino acid, sarcosine or N-methyl derivative of glycine present in muscle and other tissues collectively umbrella under a club known as metabolites [116]. Sarcosine levels help in identification of malignancy of prostate cancer cells [117], clinically localized prostate cancer and benign prostate cancer that tends to be higher for invasive prostate cancer cell lines in comparison to prostate epithelial cells [117].

It's mainly produced from glycine using glycine-N-methyl transferase making it indispensable metabolic intermediary that bolster prostate cancer cells toward metastasis and invasion [118] which facilitate the detection of aggressive prostate cancer at their aboriginal stage but still its usage as a biomarker is under consideration and further investigations are needed [119].

Androgen receptor (AR) plays a significant role in the development and progression of prostate cancer. Prostate cancers with castration resistance particularly, constitutes of many oncogenic mutations in AR such as high levels of expression, augmented copy number, alterations that affect specificity of ligand, and high level of enzyme activity involving antigen synthesis [120]. To combat these issues, a biopsy is considered for the evaluation of status of AR in tumor. Although it's a feasible approach but invasive at the same time.

##### 3.4.3.1 AR based analog16β-Fluoro-5α-dihyrotestosterone (FDHT)

To overcome the issues associated with AR a marker has been developed i.e. 16β-Fluoro-5α-dihyrotestosterone (FDHT) for imaging of tumors is an analog of 5α-dihydrotestosterone (DHT) with primary intra-prostatic structure of androgen [121]. In an animal study, it has been reported among various analogs of fluorinated, uptake of ^18^F-FDHT in the prostate was hindered by co-administration of cold testosterone and resulted in the greatest quantities of non-metabolized radiolabeled ligand in blood up to 45 min post-injection. Therefore, ^18^F-FDHT seems to bind exactly to AR *in vivo.* Hence a noninvasive approach to study tumors [122]. In another study, ^18^F-FDHT has been used for the imaging of breast cancer successfully [123].

## 4 Imaging approaches

The main imaging techniques for majority of the tumor detection are nuclear imaging, optical imaging and magnetic resonance imaging (MRI) along with that biosensors (electrochemical, nanoparticle implemented) and electro-chemi-luminescence immuno assay are also used. All the methods have their own pros and cons including sensitivity, 3-D resolution, chronological resolution, cost, and level of tissue penetration (Table 2).

**Table 2:** Diagnostic techniques and their characteristics.

Technique	Cancer	Nature of detection	Detection limit	Significance	Limitations
Computed Tomography	Almost all types of cancer (Colorectal and lungs) and tumor infection especially Bone tumors (conventional method)	X-rays	10^−10^- 10^−11^ M.	Helps in generating multiplanar reformatted imaging. Eliminates the superimposition of images of structures outside the area of interest.	Only provide information about structural Characteristics
PET	Almost All cancers	Nuclear imaging (Radioisotopes)	10^−11^- 10^−12^ M.	High sensitivity, Refined, localization, Quantification	Expensive, Qualified staff required
SPECT	Colorectal, Lung, Head, Neck, Breast, Gall bladder, Lymphoma	Nuclear imaging (Radioisotopes)	10^−10^- 10^−11^ M.	Detection of biochemical and physiological anomalies	Lengthy acquisition time, inefficiency in terms of modality constrains.
Optical imaging	Extra cranial tumors Lung, Melanoma, Laryngeal, Soft tissue	UV-Infra red. Es-Light photons.	10^−12^ M.	Cost effective, Widely available Cancer regulation, Therapeutic response, O_2_ saturation measurement	Surfaced penetration, Absorption and scattering
Magnetic resonance imaging	Brain, Head, Neck, Malignant cells	Radio waves. Magnetic waves	10^−3^- 10^−5^ M.	Robust imaging technique, Exceptional contrast	Time, Expensive.
Nanotechnology	Brain, Lungs, Colon, Esophageal, Bladder, Prostate	Nanoparticles	Depending among diagnostic technique incorporated	Highly sensitive, Stability	Under trials,

### 4.1 Computed tomography

Computed tomography (CT) or Computerized axial tomography (CAT) is an imaging procedure that use an extraordinary X-ray equipment to create scans, detailed pictures and areas inside the body (1). The term *tomography* comes from the Greek words *tomos* (a cut, a slice, or a section) and *graphein* (to write or record). By the help pf computer programs two types of pictures could be generated and visualized (2D and 3D) and the series of pictures produced look like a loaf of sliced bread that could be looked individually (2D) or as a whole (3D). CT machines have been modernized to give better scans and advanced results like Helical CT, multi-slice CT or multidetector CT scanners which allow more slices to be imaged in a shorter period of time [124]. CT could be used in cancer in many different ways like to detect abnormal growths, diagnose the presence of a tumor, provides information about the stage of cancer, to determine whether a cancer is responding to treatment and to detect the recurrence of a tumor. Apart from Cancer, CT is also widely used for the detection of atherosclerosis, spinal conditions, abscesses, inflammatory diseases injuries of head, internal organs and skeletal systems. It can be a lifesaving tool in diagnostics [125].

### 4.2 Positron emission tomography (PET)

Positron emission tomography (PET), is a nuclear imaging approach mainly used in oncology. Mallinckrodt Institute of Radiology at Washington University invented PET in the mid-1970s and approved as beneficial tool for research in neurology and cardiology. It's an expensive modality, with more than a million-dollar PET scanner, equipment and highly qualified staff to generate the radiopharmaceuticals used for PET imaging.

The working principle of PET imaging is the tagging of small, organically significant molecules, such as sugars, amino acids, nucleic acids, receptor-binding ligands. When these tracers experience radioactive deterioration, their places can be monitored by the PET scanner. By imaging the sequential dispersal of these labeled composites physiologic maps are developed [126]. PET exploits positron emitters such as ^11^C and ^18^F being most common, detects γ rays as an outcome from positron/electron annihilation. PET is capable of imaging at least of 10^−11^–10^−12^ M of a molecular probe [41]. The development of PET and computed tomography (CT) has allowed the comparatively little three-dimensional resolution of radioactive molecules images to be bonded to a high resolution functional CT image, consequently refining localization, quantification, and sensitivity [127].

### 4.3 Single photon emission computed tomographic (SPECT) imaging

Single Photon Emission Computed Tomography (SPECT) is also a nuclear imaging approach used to plot physiological and biological mechanisms in humans and animals after the administration of radiolabeled tracers. SPECT radioisotopes (^123^I, ^111^In, and ^99m^Tc, are most common) deterioration take place via capturing of electron and/or gamma emission. An exceptional benefit of PET and SPECT imaging is, their ability to detect biochemical and physiologic anomalies related to disease before the emergence of functional changes which can be envisaged by standard imaging modalities such as CT and MRI [128]. Although, the spatial resolution of MRI and CT is considerably higher than that of SPECT but SPECT has higher sensitivity of detection than former two, particularly structural modalities. Also, can detect quantity of a tracer molecule in picomolar or nanomolar scale [129]. SPECT exploit the tracer principal for the detection of physiological abnormality or disturbed biochemical process.

### 4.4 Optical imaging

Optical imaging technique is referred as an *in vivo* imaging of light photons with charge-coupled device (CCD) cameras. CCD cameras permit the imaging of light inside and outside of the visible range (ultraviolet (UV) and near infrared). A light emits i.e. fluorescent, once an electronically excited molecule changes from its ground state, becomes a source optical imaging. Advantages of using optical imaging are it is both cost effective and widely available. Most commonly used optical imaging techniques are; bioluminescence and fluorescence intensity imaging [41].

### 4.5 Magnetic resonance imaging (MRI)

Third technique used for imaging of tumors is, magnetic resonance imaging (MRI), is a robust imaging approach providing exceptional soft tissue contrast. When sample is positioned within an intense magnetic field the proton spins line up to produce a variety of dispensation dipoles positioned in parallel to the key magnetic field route. When a certain pulse of radiofrequency is directed, the targeted dipoles are displaced. Once pulse has been turned off, the dipoles relax to their normal state. T1 (longitudinal relaxation) and T2 (transverse relaxation) are the times to create MR images which highlight diverse anatomical structures and fluids. Metals with magnetic moments (Gd^3+^, Mn^2+^, Fe^3+^) are effective contrast agents for the MRI. MRI is the least sensitive, requiring 10^−3^–10^−5^ M metals as a molecular probe for detection [41].

### 4.6 Nanotechnology and tumor imaging

Nanotechnology has appeared as a significant platform for numerous of biological applications including medicine and diagnostics [130]. Although conventional molecular imaging techniques are available as described in previous section, but due to certain limitations such as poor spatial resolution, low sensitivity, or poor signal penetration through tissues making them less sensitive for tumor detection. Therefore, nanoparticles have great potential to overcome such obstacles and accurate diagnosis of cancer through accumulation and targeting approaches and are highly sensitive and stable [131].

Nanoparticles are efficientintransfer of numerous probes for molecular imaging for early-stage cancer detectionvia multiple imaging techniques such as PET, MRI, CT, and ultrasonography (US) [132]. In lung cancer mouse model study successfully used PEGylatednano-liposomal imaging agent containing indocyanine green (ICG) and iohexol, for real-time near infrared (NIR) fluorescence and computed tomography (CT) image-guided surgery [133]. PET imaging and plasmonic nanoparticles are used to image and monitor the photo thermal therapy response in mouse model [134].

## 5 Conclusion

A characteristic feature of cancer is an altered metabolism and driver of cancer progression. Biochemical pathways including glycolysis, glutaminolysis, TCA cycle and fatty acid synthesis are augmented in cancer cells as compared to normal ones. Examining and comprehensive understanding the alternations in cancer metabolism *in vivo* significantly enhances not only diagnosis but also therapy of cancer. Warburg, who observed the increased glycolysis and production of lactate in cancer vs. normal cells and hypothesized that metabolic processes in cancer tissues differ from that of normal healthy ones. This key demonstration led the basis of cancer metabolism and development of various imaging techniques of cancer such as MRI, PET, SPECT and optical imaging approaches. These imaging approaches scrutinize altered metabolic processes through certain metabolic markers whose expression varies in tumor cells as compared to normal cells. Determination of potential biomarkers and novel therapeutic targets, and pertinent diagnostic technique for early detection of cancer that even have to potential to be amalgamated to upcoming hyped nanotechnologies either for probe designing or as diagnostic technique even under experimental stage these days. Hence, early detection of cancer and improvement of real- time monitoring of tumor response to therapy are needed to recuperate the result and improving the quality of life for cancer patients.

## Declarations

### Author contribution statement

All authors listed have significantly contributed to the development and the writing of this article.

### Funding statement

This research did not receive any specific grant from funding agencies in the public, commercial, or not-for-profit sectors.

### Competing interest statement

The authors declare no conflict of interest.

### Additional information

No additional information is available for this paper.
